# Levofloxacin based vs clarithromycin based sequential therapy in *helicobacter pylori* eradication; a randomized clinical trial 

**Published:** 2018

**Authors:** Mosayeb Moradniani, Zohre Mirbeik-Sabzevari, Soleiman Jaferian, Shiva Shafiezadeh, Mohammad Javad Ehsani Ardakani, Mehrdad Mirzaee Roozbahany, Saleh Azadbakht, Hamidreza Sherkatolabbasieh

**Affiliations:** 1 *Department of Internal Medicine, Lorestan University of Medical Sciences, Khorramabad, Iran*; 2 *Student Research Committee, Lorestan University of Medical Sciences, Khorramabad, Iran.*; 3 *Gastroenterology and Liver Diseases Research Center, Research Institute for Gastroenterology and Liver Diseases, Shahid Beheshti University of Medical Sciences, Tehran, Iran*; 4 *Department of Surgery, Lorestan University of Medical sciences, Khorramabad Iran*; 5 *Department of Pediatric Medicine, Lorestan University of Medical Sciences, Khorramabad, Iran *

**Keywords:** sequential therapy, Levofluxacin, Clarithromycin, Helicobacter pylori eradication

## Abstract

**Aim:**

This study was aimed to evaluating the efficacy of levofloxacin based sequential therapy vs clarithromycin based sequential therapy in *h.pylori *(HP) eradication.

**Background:**

Several therapeutic regimen were investigated to treat HP infection. Sequential therapy is an alternative to classic triple therapy.

**Methods:**

In this randomized clinical trial, 200 HP infected patients randomly divided into two therapeutic groups .1-Levofloxacin based sequential regimen (group A); omeprazole and amoxicillin for 7days followed by omeprazole, amoxicillin and levofloxacin for 7days. 2-clarithromycin based sequential regimen (group B): omeprazole and amoxicillin for 7days followed by omeprazole, amoxicillin and clarithromycin for 7days. HP eradication was evaluated with urea breath test with carbon 13 (UBT) 6 weeks after the end of treatment.

**Results:**

Per protocol eradication rates of group A and B were 87.6% and 76% respectively. By intention to treat analysis, eradication rate of group A and B groups were 85.1% and 73% respectively. Levofloxacin based sequential regimen was more effective than clarithromycin based sequential regimen (Pv=0.028).

Adverse events were seen in 19.6% and 15.6% in group A and B respectively. Drug compliance was 97% in group A and 96% in group B. There was no significant difference between two groups in term of adverse events (p=0.470) and compliance (p=0.651).

**Conclusion:**

Levofluxacin based sequential therapy was more effective than Clarithromycin based sequential therapy in HP eradication. The suggested Levofluxacin based sequential therapy could be an alternative therapy in area with high clarithromycin resistance. Further studies are needed to confirm these findings.

## Introduction

*Helicobacter pylori *(HP) affected 50% of the world population and results in problems such as dyspepsia, peptic ulcer disease, gastric cancer, gastric MALToma. HP is more common in developing countries (1). Treatment of HP infection is a major challenge for physicians worldwide (2). Due to the chronicity of HP infection, the simultaneous use of two or more antibiotics is required to eradicate this bacteria (3). Sequential therapy was first introduced in 2000 by Zullo *et al*(4). Sequential regimen consists of two phases in which PPI and amoxicillin are administrated for 5 to 7 days. Then in the second phase, PPI, clarithromycin and metronidazole (or tinidazole) are administrated for 5 to 7 days (5, 6). Initial studies have reported the eradication rate of HP infection very high by using sequential regimen (about 95%)(6). A recent meta-analysis revealed the eradication rate with classic sequential regime as 80.8% (7). 

Levofloxacin is an antibiotic from Fluoroquinolones group (8). Since the resistance to levofloxacin in HP strains is low (9, 10), the use of levofloxacin instead of clarithromycin in the sequential therapy could increase the effectiveness of this regime (11). Levofloxacin-based sequential therapy is a good alternative to classic sequential therapy. Majority of studies were revealed that eradication rate with this regimen is very high (7, 12, 13). The aim of this study was to compare the efficacy of levofloxacin-based sequential therapy and Clarithromycin based sequential therapy in HP eradication. 

## Methods

Study design and patients: this prospective, randomized clinical trial was conducted between October 2015 and March 2015. This study surveyed, dyspeptic patients who were referred to gastroenterology clinic of Shohaday-e- Ashayer hospital in Khorramabad (a city in west of Iran).

HP status was proven by histologic examination. Two hundred HP infected patients were enrolled in the study. Inclusion criteria were; proven positive for HP infection and be 18 years or older. Exclusion criteria included; (a) previous history of HP eradication, (b) being younger than 18 years old, (c) being allergic to the medications used in the study, (d) history of gastric surgery, (e) serious underlying disease; such as decompensated cirrhosis and Chronic renal failure and (f) pregnancy or lactation.

Intervention: Patients were randomly assigned to one of the therapeutic groups by computer-generated assignment. A research assistant managed the randomization. Eligible patients randomly received one of the therapeutic regimens.

1-Levofloxacin based sequential group (Lev-seq) received Omeprazole 20mg and amoxicillin 1g twice daily for 7 days followed by omeprazole 20mg, amoxicillin 1g, levofloxacin 500 mg twice daily for 7 days. 

2-Clarithromycin based sequential group (Cla-seq) consists of omeprazole 20mg and amoxicillin 1g twice daily for 7 days followed by omeprazole 20mg, amoxicillin 1g, Clarithromycin 500mg twice daily for 7days. Patients were given a written instruction on how to take the drugs correctly. This study was approved by the ethic committee of Lorestan University of Medical Science in Lorestan province, Khorramabad. The study was registered in Iranian Registry of Clinical Trials (IRCT2015082323736N1).

Primary Outcome (helicobacter pylori eradication): Primary outcome of this study was helicobacter pylori eradication rate. We invited all patients to take a 13C-urea breath test (UBT), six weeks after completing the treatment. UBT was done with 75 mg of 13C-urea in fasting state solved in 100 ml of orange juice. The 13C in the expired air was measured 20 minutes later, using an infrared spectrophotometer (PY test, Kimberly-Clark, USA). 

Secondary outcome (compliance & side effect): All patients were visited by physician, one week after the onset of drug administration and the end of treatment period. Drug compliance and adverse events were evaluated by physician and were recorded. Drug compliance was evaluated by pill counts. Good compliance was defined as taking more than 90% of the total medication. Side effects and safety of therapeutic regimes were evaluated by questionnaire .

Statistical Analysis: Eradication rate was calculated as the percentage of patients whose HP infection was negative at the end of the study .The eradication rate was determined by intention-to-treat (ITT) and per-protocol (PP) analyses. The SPSS 21.0 statistical software was used for statistical analysis. Statistical analysis of the results was performed using a Chi-square test, Student’s t-test. Multiple regression analysis were done to identify factors that might have influence on eradication rate of each therapeutic regimens.

**Figure 1:**
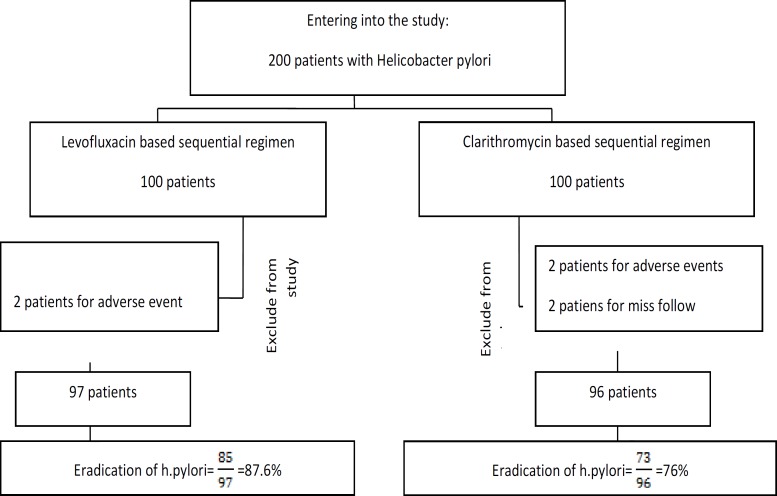
patients design

## Results

Two hundred HP-infected patients entered the study. The patients randomly assign in two therapeutic groups. All subjects were included in the ITT analysis for HP eradication. Among the subjects, three patients with incomplete follow-up and four patients because of severe side effects excluded from study (figure1).

Finally, 193 patients completed the study. Levofloxacin based sequential (Lev-seq) group included 51 women and 46 men. Clarithromycin based sequential (Cla-seq) group included 48 women and 48 men. Mean age of Lev-seq and Cla-seq groups were 40.2± 11.8 years and 42±13.02 years respectively .Demographic and clinical characteristics of patients are summarized in table 1 (table1). There were no significant differences with age, gender, education level and smoking between two groups (p˃0.05).

**Eradication rate of** ***Helicobacter pylori***

The eradication rates in Lev-seq group was 87.6% (95%CI) by per protocol analysis and 85% (95%CI) by intention to treat analysis. Per protocol eradication rate of Cla-seq group was 76% (95%CI), and in intention to treat analysis was 73% (95% CI). Both ITT and PP analysis showed that levofloxacin based sequential regime was more effective than clarithromycin based sequential regime. This difference was statistically significant (p-value=0.028).

**Table 1:** Baseline demographic and clinical characteristics of patients

Cla-seq group N (%)	Lev-seq group N (%)		Variables
		**Sex**	
48(50%)	46(47.7%)		Male
48(.50%)	51(56.6%)		Female
			**Age(year)**
13.02± 42	11.8±40.2		Mean±SD
(19.8%)19	(18.6%)18		18-30
(30.2. %)29	(34%)33		31-40
(22.9%)22	(28.9%)28		41-50
(27.1%)26	(18.6%)18		≥51
			**smoking**
16(16.7%)	16(16.5%)		yes
80(83.3%)	81(83.5%)		no
			**education**
21(21.9%)	19(18.5%)		illiterate
10(10.4%)	13(13.4%)		Primary
16(16.7%)	17(17.3%)		Secondary school
22(22.9%)	21(21.6%)		diplomas
27(28.1%)	27(29.2%)		College education

The odds ratio for eradication of HP with Lev-seq group compared with Cla-seq group was 2.1 (95% CI 1.1-4.0) )table 2).

**Adverse events and compliance**

 Nausea (13.4%) and anorexia (8.2%) were the most common adverse events in lev-seq group. The most common adverse events of Cla-seq group were anorexia (9.4%), abdominal pain and nausea (both 8.3%). No significant difference was seen between two groups in term of adverse events. (p=0.470) 

Overall, four patients stop the treatments due to sever adverse events, including two patients at each group. Three patients withdrew the study, because they were lost to follow. 97 patients in Lev-seq group and 96 patients in Cla-seq group were taking more than 90% of total medication. Treatment compliance in lev-seq groups and Cla-seq were 97% and 96% subsequently. There was no significant difference between two groups (p˃0.05) (Table3).

**Factors affecting the eradication rate**

Analysis the variables that may affect eradication rate was done by multiple regression analysis. In the multivariate analysis, the efficacy of levofloxacin and clarithromycin based sequential therapy were not influenced by the gender, age, smoking and education level. We did not evaluate the antibiotic resistance in this study. So the therapeutic regime was the only factor that has an effect on the eradication rate.

## Discussion

This randomized clinical trial showed that the eradication rate of HP with levofloxacin based sequential regime is more than clarithromycin based sequential regime. Eradication rate of lev-seq therapies 87.6% and 85.1% by PP and ITT analysis respectively. Per protocol eradication rate of cla-seq therapies 76% and eradication rate of cla-seq therapy by ITT analysis is 73 %. There was no statistically significant difference between compliance and drug side effects in both groups. Rising the antibiotics resistance, especially clarithromycin and reduction of HP eradication rate with classic triple regimen, led to introduction of safe and more effective therapeutic regimes (2, 8). Sequential therapy includes PPI and amoxicillin for 5 to 7 days followed by PPI, clarithromycin and metronidazole (or tinidazole) for 5 to 7 days (4). HP eradication rate with classic sequential therapy in the early studies was very high (about 95%)(4, 5). However, recent studies showed that eradication rate by classic sequential therapy is decreasing (14). In a recent meta- analysis, the overall rate of HP eradication with this regime was about 83% (15). The results of our study suggested that lev-seq therapy is more effective than Cla-seq therapy. Few studies have compared these two sequential regimes. In a randomized clinical trial in Italy, eradication rate of lev-seq regime was higher than 95% and subsequent eradication rate of Cla- seq regime was 80.8%(14). Other study conducted in Spain showed that the eradication rate of cla-seq regime was 76.5% and the eradication rate of lev- seq regime was 82.5% (13).

Our results are similar to those obtained from these studies. Other studies in this field also show the preference of levofloxacin based sequential regime. A recent meta-analysis confirmed the superiority and efficacy of levofloxacin-based sequential therapy on classic sequential therapy (7). The main reason for the effectiveness of lev-seq therapy is low rate of resistance to levofloxacin, and high levels of resistance to the clarithromycin (12, 13, 16). Most studies in different countries showed that the rate of resistance to levofloxacin in HP strains is low (9, 10). In Iran also similar to other countries in the world, levofloxacin resistance rate is low as 5.3% (10). The results of our study shows that the eradication rate with cla-seq regime is suboptimal (less than 80 percent) and also lower, compared to other studies. Our results are similar to those obtained from other studies. The main reason for the low rate of eradication is the high level of resistance to clarithromycin. An alternative explanation for the low rate of eradication with classic sequential regime could be the difference in the type of used PPI in the study and use of tinidazole instead of metronidazole in some studies. Some studies have suggested that due to the high level of metronidazole resistance and longer half-life of tinidazole, the use of tinidazole in regimen may increase the success rate of treatment (17). Recent studies have demonstrated that the use of new generation PPI such as esomeprazole and rabeprazole leads to achieve higher rates of eradication (18-20). Levofloxacin based sequential regime is a promising strategy in the treatment of HP infection. Some studies have proven that in-vitro usage of levofloxacin has significant effect on HP strains (21) as well as the resistance to levofloxacin much less than resistance to other antibiotics. In addition, other studies have shown that levofloxacin is effective in the treatment of HP strains that are resistant to clarithromycin and metronidazole (22). Our study had some limitations. The main was that, we have not been assessed the susceptibility of HP strain to antibiotics and antibiotic resistance rates. However, in this study we used the data obtained from another study that was done in our country. Clarithromycin resistance rate in Iran is 22.4%(23). Another limitation of this study is that it has been conducted in one center and on a small number of samples and the result cannot be generalized to other centers of the country. Many factors may have an impact on the eradication rate of HP. Based on the results of our study, factors such as age, sex, smoking, level of education and the underlying disease had no effect on eradication rate. The result of our study is similar to other studies (7, 14). In our study there was no significant difference between two groups in term of adverse events and medication compliance. Adverse events rate in lev-seq regime was 19.6% and in Cla- seq regime was reported as 15.6%. The most frequent side effects in lev-seq group were nausea and anorexia. Abdominal pain and loss of appetite were the most common side effects in the Cla- seq group. 

**Table 2.** Eradication rates with levofoxacin and clarithromycin based Sequential regime

		Helicobacter pylori status after treatment		Total	P value	OR, CI(95%)
		not eradicated	eradicated			
Lev-seq group	Number	12	85	97	0.028	2.1(1.10-4.09)
	Percent	12.4	87.6	100		
Cla-seq group	Number	23	73	96		
	Percent	24	76	100		

**Table 3.** Adverse events in the levofluxacin and clarithromycin based sequential therapy

P value	CI(95%)	OR	Cla-seq group N (%)	Lev-seq group N (%)	Variables
0.084	0.856-1.04	1.608	8(8.3%)	13(13.4%)	Nausea
0.505	0.254-4.43	1.485	2(2.10%)	3(3.1%)	Vomiting
0.494	0.171-0.322	0.742	4(4.2%)	3(3.1%)	Diarrhea
0.492	0.327-2.29	0.866	8(8.3%)	7(7.2%)	Abdominal pain
0.354	0.205-4.78	0.990	3(3.1%)	3(3.1%)	Constipation
0.490	0.354-2.18	0.880	9(9.4%)	8(8.2%)	Anorexia
0.261	-	-	0(0%)	2(2.1%)	Rash
0. 09	0.614.4.89	2.992	2(2.1%)	6(6.2%)	Headache
0.492	.296 -2.95	0. 848	7(7.3%)	6(6.2%)	Bad taste
0.115	0.734-1.99	1.09	1(1.1%)	2(2.1%)	Patient who withdrew
0.651	0.385-3.13	1.78	96(100%)	97(100%)	Good compliance

The results of our study and other studies are consistent (12, 13, 24), but the type of side effects were different (7, 13, 14). The main reason maybe the differences in genetic and environmental factors. In the study of romana *et al*, the most common side effects are abdominal pain and diarrhea in the first 5 days and bad oral taste at the second 5-day of administration of levofloxacin and also one case of severe pain in tendon which was not reported in the present study (13).

The eradication rate of clarithromycin based sequential regime is suboptimal (less than 80%). Levofloxacin based sequential therapy is much more effective than clarithromycin based sequential therapy. Further studies are needed with larger samples and multicenter in different parts of the country for further evaluation of this regimen

## References

[1] Moradniani M, Mirbeik-Sabzevari Z, Bahmani M, Azadbakht S, Jaferian S, Sherkatolabbasieh H (2017). Comparison of 7–Day Concomitant Therapy Regimen Versus Classic Triple Therapy Regimen In Helicobacter Pylori Eradication: A Randomized Clinical Trial. Int J Pharm Sci Res.

[2] Chey WD, Leontiadis GI, Howden CW, Moss SF (2017). ACG Clinical Guideline: Treatment of Helicobacter pylori Infection. Am J Gastroenterol.

[3] Selgrad M, Bornschein J, Malfertheiner P (2011). Guidelines for treatment of Helicobacter pylori in the East and West. Expert Rev Anti Infect Ther.

[4] Zullo A, Rinaldi V, Winn S, Meddi P, Lionetti R, Hassan C (2000). A new highly effective short-term therapy schedule for Helicobacter pylori eradication. Aliment Pharmacol Ther.

[5] Jafri NS, Hornung CA, Howden CW (2008). Meta-analysis: sequential therapy appears superior to standard therapy for Helicobacter pylori infection in patients naive to treatment. Ann Intern Med.

[6] Vaira D, Zullo A, Vakil N, Gatta L, Ricci C, Perna F (2007). Sequential Therapy versus Standard Triple-Drug Therapy for Helicobacter pylori EradicationA Randomized Trial. Ann Intern Med.

[7] Kale‐Pradhan PB, Mihaescu A, Wilhelm SM (2015). Fluoroquinolone Sequential Therapy for Helicobacter pylori: A Meta‐analysis. Pharmacotherapy: Pharmacotherapy.

[8] Malfertheiner P, Megraud F, O'morain CA, Atherton J, Axon AT, Bazzoli F (2012). Management of Helicobacter pylori infection—the Maastricht IV/Florence consensus report. Gut.

[9] De Francesco V, Giorgio F, Hassan C, Manes G, Vannella L, Panella C (2010). Worldwide H. pylori antibiotic resistance: a systematic. J Gastrointestin Liver Dis.

[10] Moradniani M, Mirbeik-Sabzevari Z, Bahmani M, Azadbakht S, Jaferian S, Sherkatolabbasieh H (2017). Comparison of 7-day concomitant therapy regimen versus classic triple therapy regimen in Helicobacter pylori eradication: A randomized clinical trial. Int J Pharm Sci Res.

[11] Sherkatolabbasieh H, Shafizadeh S, Azadbakht S, Moradniani M, Maleki H, Jaferian S (2017). Levofloxacin-based sequential therapy versus classic triple therapy in Helicobacter pylori eradication: A randomized clinical trial. Biomedical Research and Therapy.

[12] Liou JM, Chen CC, Chen MJ, Chen CC, Chang CY, Fang YJ (2013). Sequential versus triple therapy for the first-line treatment of Helicobacter pylori: a multicentre, open-label, randomised trial. Lancet.

[13] Romano M, Cuomo A, Gravina AG, Miranda A, Iovene MR, Tiso A (2010). Empirical levofloxacin-containing versus clarithromycin-containing sequential therapy for Helicobacter pylori eradication: a randomised trial. Gut.

[14] Molina‐Infante J, Perez‐Gallardo B, Fernandez‐Bermejo M, Hernandez‐Alonso M, Vinagre G, Duenas C (2010). Clinical trial: clarithromycin vs levofloxacin in first‐line triple and sequential regimens for Helicobacter pylori eradication. Aliment Pharmacol Ther.

[15] Gatta L, Vakil N, Leandro G, Di Mario F, Vaira D (2009). Sequential therapy or triple therapy for Helicobacter pylori infection: systematic review and meta-analysis of randomized controlled trials in adults and children. Am J Gastroenterol.

[16] Qian J, Ye F, Zhang J, Yang YM, Tu HM, Jiang Q (2012). Levofloxacin‐containing Triple and Sequential Therapy or Standard Sequential Therapy as the First Line Treatment for Helicobacter pylori Eradication in China. Helicobacter.

[17] Moradniani M, Firouzi M, Baharvand SP, Maleki H, Ghaderi S, Sherkatolabbasieh H (2018). A randomized clinical trial; co levofloxacin based sequential based sequential versus triple. Helicobacter pylori eradication.

[18] Villoria A, Garcia P, Calvet X, Gisbert J, Vergara M (2008). Meta‐analysis: high‐dose proton pump inhibitors vs standard dose in triple therapy for Helicobacter pylori eradication. Aliment Pharmacol Ther.

[19] McNicholl A, Linares P, Nyssen O, Calvet X, Gisbert J (2012). Meta‐analysis: esomeprazole or rabeprazole vs first‐generation pump inhibitors in the treatment of Helicobacter pylori infection. Aliment Pharmacol Ther.

[20] Choi HS, Park DI, Hwang SJ, Park JS, Kim HJ, Cho YK (2007). Double‐Dose, New‐Generation Proton Pump Inhibitors Do Not Improve Helicobacter pylori Eradication Rate. Helicobacter.

[21] Dubreuil L, Devos J, Beerens H, Romond C (1988). In vitro activity of an ofloxacin-metronidazole combination against anaerobic bacteria Kinetics of the action of metronidazole against Bacteroides fragilis. Pathol Biol.

[22] Wu W, Yang Y, Sun G (2012). Recent insights into antibiotic resistance in Helicobacter pylori eradication. Gastroenterol Res Pract.

[23] Fallahi GH, Maleknejad S (2007). Helicobacter pylori culture and antimicrobial resistance in Iran. Indian J Pediatr.

[24] Lee H, Hong SN, Min BH, Lee JH, Rhee PL, Lee YC (2015). Comparison of efficacy and safety of levofloxacin-containing versus standard sequential therapy in eradication of Helicobacter pylori infection in Korea. Dig Liver Dis.

